# Diurnal Body Temperature and Rate of Passage of Loggers in Lions

**DOI:** 10.3390/ani10081388

**Published:** 2020-08-11

**Authors:** Ted Friend, Giulia Corsini, Vincent Manero, Raffaella Cocco

**Affiliations:** 1 Department of Animal Science, Texas A&M University, College Station, 3785 W. Villa Maria Rd., Bryan, TX 77807, USA; 2 Independent Researcher, Peterborough PE1 4DG, UK; giulia.corsini@outlook.com; 3 Independent Researcher, Le Coine, 31352 Agiez, Switzerland; vmanero@bluewin.ch; 4 Department of Veterinary Medicine, University of Sassari, 07100 Sassari, Italy; rafco@uniss.it

**Keywords:** lions, body temperature, rate of passage, loggers, circadian rhythm

## Abstract

**Simple Summary:**

It is assumed that all mammals have circadian rhythms in their body temperatures, but that has never been described in African lions. The disruption of circadian rhythms can indicate poor animal welfare. Thirteen miniature temperature data loggers were fed to 11 lions, and reliable data were obtained for seven of the lions. Finding the small loggers after they passed through the lions was a challenge because the lions could move between their bedded and heated dens and an outside exercise area. The loggers passed through the lions in an average of 22 h, so future research using the non-invasive method of feeding temperature loggers is limited to that time frame. A highly significant diurnal pattern in body temperature was found, with temperatures being 1.14 °C higher in the afternoon than in the morning. Hence, alteration of body temperature rhythm could be useful in assessments of animal welfare.

**Abstract:**

The documentation of diurnal patterns in body temperature in lions could be important because disruption of circadian patterns can be a useful measure of distress. This study quantified changes in body temperature of seven African lions *(Panthera leo)* at 5 min intervals during cold conditions from noon until the ingested body temperature loggers were expelled the next day. Thirteen loggers were fed to 11 lions during their daily noon feeding, while ambient temperatures were also recorded using six data loggers. The lions had continuous access to their dens and exercise pens during the day but were restricted to their heavily bedded dens that also contained a heat lamp from 23:00 until 08:00 the next day. Body temperatures averaged 37.95 ± 0.42 °C at 15:50, and 36.81 ± 0.17 °C at 06:50 the next day, 30 min before the first loggers passed from a lion, and were significantly different (t-test, t = 8.09, df = 6, *p* < 0.0003). The mean duration for the time of passage was 22 ± 2.69 (h ± SD), so future studies using the noninvasive feeding of temperature loggers need to consider that time frame.

## 1. Introduction

There is an absence of objective information which lion keepers, the public, and decision makers can utilize for making informed decisions regarding the care and husbandry of lions (*Panthera leo*). The existence of and factors associated with circadian rhythms have been extensively studied in many species. For example, Mortola and Lanthier [1] based their analysis on a survey of over 200 studies investigating their parameters of interest. The documentation of diurnal patterns in body temperatures of lions could be important, because a change in amplitude over 24 h [1]—disruption [2] or displacement [3], for example**—**of circadian patterns can be a useful measure of distress because many of the regulatory systems involved in stress responses are linked to circadian systems [4]. Diurnal patterns in body temperature usually consist of a rise in temperature during the daytime activity phase and a decrease in temperature during the nighttime rest phase in mammals, e.g., [1,4].

However, no body temperature data exist for lions that document their diurnal pattern in body temperature. Body temperatures were recorded overnight at 5 min intervals for tigers traveling with six different circus acts in North America [5], but activities, time of day, and time in transit varied too much for inferences regarding circadian rhythms to be made. Trethowan et al. [6] used surgically implanted temperature loggers in free ranging African lions that recorded body temperature at 5 min intervals over extended periods, but those researchers used daily averages as the experimental unit. Trethowan was not able to recover all of their implanted loggers. Similarly, surgical implantation of temperature data loggers has been used in many other species, e.g., koalas [7], but a less invasive method may be useful in certain situations.

Feeding miniature body temperature loggers to lions has welfare advantages over surgical implantation and later surgical removal, although the transit time through a lion needs to be elucidated and finding the expelled loggers can be a challenge. Although this study could not be replicated because similar climatic conditions did not occur during the window of opportunity allowing for a replication, these data have value because consistent data were obtained from six different lions. Additionally, a second body temperature logger was recovered from one lion in this study, providing a replication within one of the subjects. Because sizeable populations of big cats are rare, a behavioral study using three lions and a case report with one tiger [8], for example, have value.

The goal of this study was to describe diurnal temperature patterns in adult lions, estimate the time it takes for orally administered miniature temperature loggers to pass through adult lions, and to report on any problems arising from using orally administered temperature loggers. Although it can be inferred that diurnal patterns in core body temperature occur in lions, no documentation has been reported.

## 2. Materials and Methods

### 2.1. Subjects

The subjects used in this study were part of the company “La Favola Siamo Noi” (We Are The Fairy Tale) registered to Mrs. Valeria Afrodita Valeriu, Tagliamento 42, Quinto Vicentino 36050 (IV), Italy. The lions and tigers were inspected by the Veterinary Services ULSS 8 (Unità Locale Socio Sanitaria, Quinto Vicentino, Vicenza, Italy) in addition to local authorities. During this study, the Valeriu family had 11 lions (four mature females, one mature male lion, three young females, and three young males) and two tigers (two females). All of the family members contribute in a multitude of ways, including husbandry, training, and presenting their animals. At the time of this study, the animals were located in a field in Trecate, Italy, about 50 km West of Milan, where they had been for 4 d prior to the start of data collection.

The feeding of Thermochron temperature loggers to performing lions and tigers was approved by the Texas A&M University Animal Care and Use Committee. The technique has been used in published [5] and many unpublished studies without any adverse effects on the subjects. Because the lions in this study were privately owned, the study was also approved by the owner of the subjects, their trainers and care givers, and their consulting veterinarians.

The cats were fed between 11:00 and noon, but a portion of their meat was also fed in the form of rewards when they were trained, moved from one pen to another, or performing. The first performance was at 17:00 and the second at 21:00. The lions’ performances lasted about 9 min.

### 2.2. Housing of the Lions

**Figure 1:**
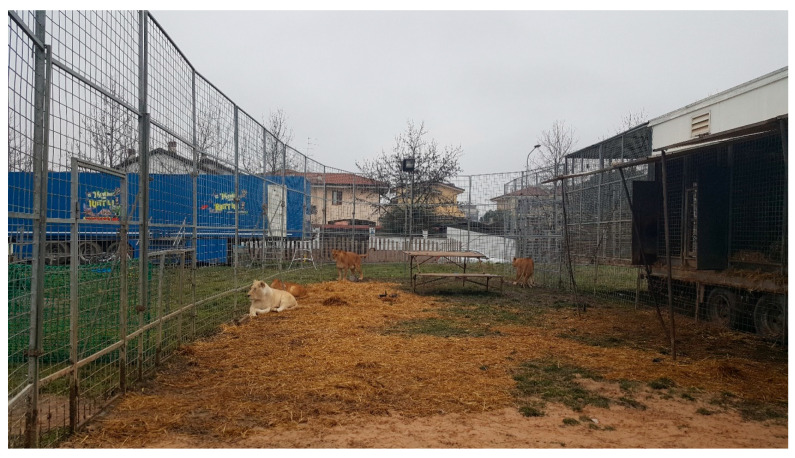
One of the exercise pens containing four of the lions used in this study. A second pen is visible in the background. The dens, which also served as travel cages, are located in the trailer to the right.

The lions (and tigers) had access to exercise pens during most of the day (Figure 1), and were rotated through the connected exercise pens and cages to allow for daily cleaning of the pens and dens, training sessions, enrichment sessions, feeding, and performances. The lions almost always had access to their home dens from their exercise pens. Although there can always be variation in how lion exhibitors may set up, depending on the venue, equipment, and weather, this exercise pen scheme has been common since the 1990s. Kiley-Worthington [9] has a description of early exercise pens used in England in 1990. Krawczel et al. [10], Nevill and Friend [11], and Nevill et al. [12] further reported on the utilization of exercise pens by circus tigers.

Because the caregivers of the lions judged conditions to be cold, but also for security reasons, the sides of the dens were closed after the last performance at night. Access to the exercise pens was also prevented until the next morning at 08:00. Each den was deeply bedded with a mixture of straw and sawdust, and also had supplemental heating in the form of a radiant heat lamp. The lions and two tigers were maintained in groups of up to four individuals, except for the adult male lion, who was housed singly to avoid him breeding with the females (Table 1). The dimensions of the exercise pens and dens varied with the size of the group (Table 1).

### 2.3. Body Temperature

Thirteen calibrated Thermochrons (also known as “iButton” temperature loggers, maximintegrated.com, Model DS1921 “H” with precision of 0.125 °C, range 15–46 °C) programmed to record temperatures at 5 min intervals were fed to 11 of the Valeriu lions, commencing a little before noon on 3/6/2018. Although the Valeriu Family also had two tigers, only their lions were used in this study to reduce possible sources of variation if a combination of tigers and lions were used. Additionally, studies have been published using performing tigers in the USA [5]. Calibration and functioning of the loggers was checked by allowing the loggers to stabilize at room temperature and run for 30 min. Data from the loggers were then downloaded, and any loggers that differed from the group mean by more than 0.125 °C (one temperature increment) were discarded.

**Figure 2:**
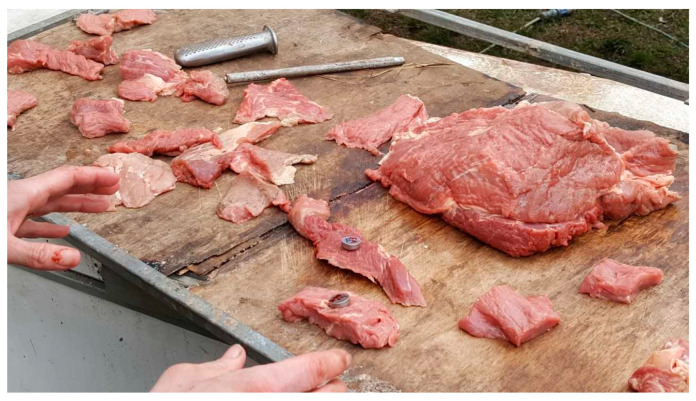
Preparing the Thermochrons to be fed to the lions. Each Thermochron was inserted into a small slit that was made in the chunks of meat to help insure the Thermochron was ingested.

**Figure 3:**
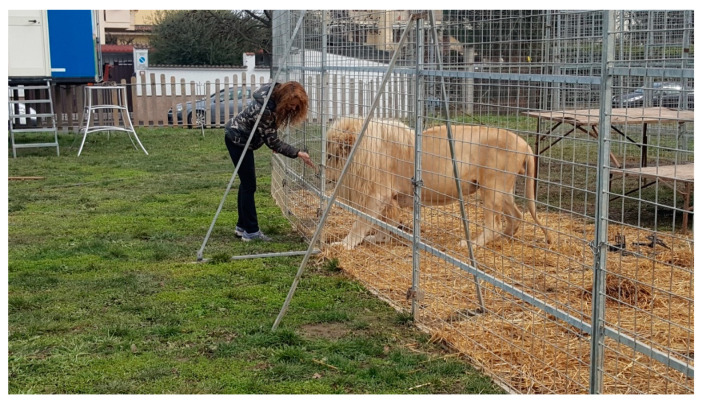
Feeding a body temperature logger to Zeus, the adult male.

The lions were usually fed pieces of meat and rewarded with pieces of meat, making it easy to slip the Thermocrons into a pocket cut into a piece of meat during their regular noon feeding (Figure 2). As is typical for big cats, the lions swallowed these pieces of meat without chewing (Figure 3). After initially feeding each of the subjects a single logger, two of the lions were fed a second logger in the hopes of getting an estimate of how closely the two loggers compared when traveling through the digestive system of a lion, and to increase our chances of finding at least one logger from those lions. From our prior research with tigers [5], we expected the Thermochrons to start being expelled the next morning when the cats became active and had a bowel movement. Finding the small expelled Thermochrons was particularly challenging because there was a large amount of bedding in the dens where the lions spent the night and the cats also had access to relatively large exercise pens starting at 08:00. Soiled bedding and droppings were stacked outside the dens and the exercise pens until the material was searched for the Thermochrons. The floors of the dens and exercise pens were also searched. The Thermochrons are too small to be detectable using conventional metal detectors.

**Figure 4:**
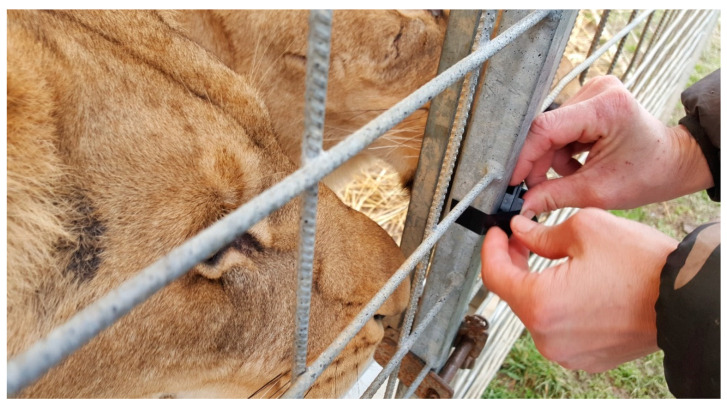
Attaching a Thermochron to the outside of Group 3′s exercise pen to record ambient temperature. The logger was not placed directly against the steel cage.

Environmental temperatures were recorded using six Thermochron/iButton model “G” temperature data loggers (maximintegrated.com) that were calibrated following the same procedure as the body temperature loggers. The model G records over a lower temperature range that the model H, −30 to +30 °C, but has less precision, 0.5 °C, because of the expanded temperature range. Two loggers were placed in the shade on the outside of two of the exercise pens used by the lions (Figure 4). If a logger was mounted in contact with a surface, 3 mm of foam insulation was placed between the back of the logger and the surface on which it was mounted so the logger could more accurately sense air temperature. Two loggers were also each placed in a “black globe” to give a relative measure of any sunshine. The globe was a table tennis ball painted flat black to better absorb any radiant heat from the sun. The black globes were mounted where they were fully exposed to the sky; one was on the top of the tunnel that led the lions into the performance tent and the second on a support outside Group 3′s exercise pen. Unfortunately, there was no sunshine during the period of the study, so the black globes were identical to our “outside” temperatures, which is what would be expected. Another Thermochron G was mounted inside Zeus’ den, the single adult male, and another was mounted inside Group 4′s den, approximately 1 m off the floor. In order to keep the lions from investigating the Thermochrons, they had to be mounted just outside of the dens. Because directional radiant heat was being used, the den temperature loggers were cooler than what the lions could experience when they moved under the heaters, so these readings need to be interpreted with caution.

## 3. Results

**Figure 5:**
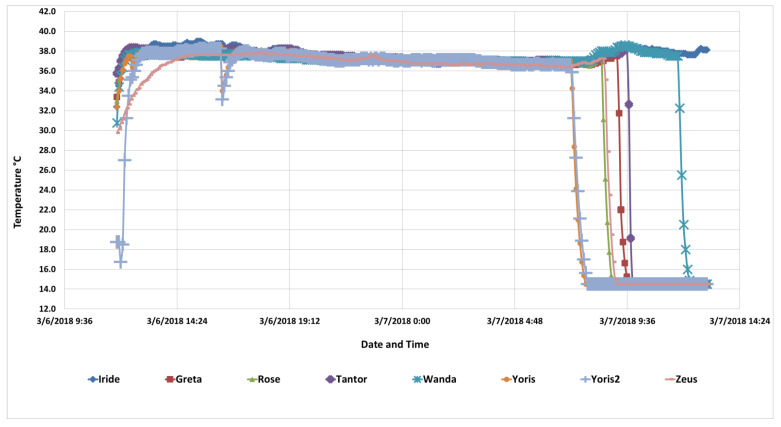
Body temperature data for the seven lions (two loggers were recovered for Yoris) commencing as the loggers were fed to the lions at noon, and terminating at 13:00 the next day. The thick blue and teal lines are because multiple temperatures were essentially identical. Iride’s body temperature data (enhanced black line) that extended until her logger was expelled at 14:50 is not shown after 13:00.

**Figure 6:**
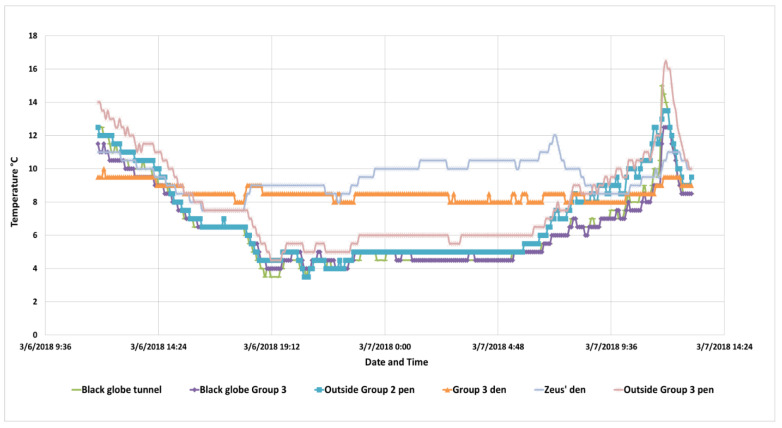
The six environmental temperature loggers show that the temperatures recorded within the dens remained several degrees warmer at night than outside temperatures, even though the loggers were outside of the focus of the infrared heaters within each den.

Nine of the ingested loggers were recovered, a good rate of recovery considering that the subjects had a lot of bedding and access to the exercise pens. Unfortunately a young boy whose parents were with the circus picked up one of the expelled loggers and held it against a magnet he had in his pocket to see if it was magnetic, erasing all the data in the logger. Hence, data from eight ingested loggers (Figure 5) are reported along with the six environmental loggers (Figure 6). The plots in the figures commence as the last of the Thermochrons was being fed so that readers can see the responsiveness of the loggers.

Several temperatures started off low, which reflects the temperature of the meat prior to the loggers being fed to the lions. After all the subjects received their initial loggers, the two loggers that were being saved in the event of a problem were fed to two lions who had already been fed a logger to yield replicates. Yoris’ second logger, Yoris2, was the last logger fed to a lion, and it shows how quickly the Thermochrons can respond to being ingested. Zeus’ logger was slower to reach body temperature than the others. That was not unexpected because a flap was cut into each piece of meat into which each Thermochron was inserted. We hypothesize that Zeus’ Thermochron remained in the meat longer than with the other lions, and the cold meat insulated the logger from quickly reaching body temperature. This logger also responded in a similar manner as the other loggers did when they were expelled, reinforcing the contention that his logger was fully functional.

Yoris was the only lion from which we recovered two functional loggers, and the temperatures those loggers recorded were essentially identical. Yoris is particularly interesting because he drank some water at 16:20 when both Thermochrons were still high enough in his gastrointestinal tract that the temperature dropped in that region of his tract. The loggers responded within one reading to the cold water. It took six readings, or about 30 min, for the Thermochrons to fully return to core temperature. Both loggers also passed from Yoris at the same time, the same bowel movement, although there was a difference of approximately 20 min between when the loggers were fed.

The body temperatures of all the lions were within the normal range (e.g., Trethowan et al. [4] determined mean daily temperatures of 37.7 ± 0.1 °C male; 37.8 ± 0.1 °C female over summer (December–February), and 37.3 ± 0.3 °C male; 37.5 ± 0.5 °C female over winter (June–August)). A diurnal pattern is evident in Figure 5 with body temperatures being higher during the day and evening, and lower late night and early morning. Body temperatures for these lions averaged 37.9 ± 0.42 °C at 15:50, 30 min before Yoris drank, and averaged 36.8 ± 0.17 °C at 06:50, 30 min before the first loggers passed from a lion (Yoris), and were significantly different (paired sample t-test, t = 8.09, df = 6, *p* < 0.0003). A trend for body temperature to increase is visible near the time when Geta and Tantor’s loggers passed (07:50–09:50), when the lions became active for a short period after the shutters on their dens were opened and they had access to their exercise pens.

The precipitous drop in the body temperatures that occurred in the morning of 3/7/2018 was when the loggers passed from each lion and cooled to the lower limits of the model H Thermochron, 15 °C. The time it took each expelled logger to reach the 15 °C floor ranged from 10 to 35 min, or two to seven recordings. The rate of decrease was likely a function of the amount of warm fecal matter and bedding that surrounded the logger as it lay in the cage or exercise pen. However, quantifying and analyzing each bowel movement and immediately recovering the expelled loggers would disrupt the lions, and was beyond the scope of this study.

The mean duration for the time of passage was 22 ± 2.69 (h ± SD), with a range of 19.2 to 27 h. Most of the cats passed their loggers when they became active in the morning and had their first bowel movement. Iride, however, did not pass her logger for another 3.0 h after the last lion (Wanda) in the group, shown in Figure 5, passed her logger. The plot of Iride’s temperature data, shown in Figure 4, had to be terminated early because including it would have compressed the horizontal scale so much that other important data could not be shown. Her body temperature did remain relatively consistent during that period, ranging between 38.25 and 38.75 °C.

Two slightly different trend lines are visible in the six Thermochrons measuring environmental temperatures (Figure 6). The temperatures obtained within the two dens (“Group 3 Den” and “Zeus’ Den”) remained relatively consistent and were 3 to 5 °C warmer than the other outside temperature readings during the coldest time of the night. The lowest set of horizontal plots are the two “outside” loggers and the two outside “black globe” loggers. The day of the observations was overcast so the black globe temperatures were similar to what the bare “outside” loggers recorded. The outside low for that night was 4 °C, which is above temperatures lions in the wild experience in their historic range. The outside temperature did warm as the clouds lessened for a short period at noon.

## 4. Discussion

The performances were a form of play, and varied because they were based on what a particular lion wanted to do in response to some coaxing, and were reinforced with positive rewards (pieces of meat and caresses). If a lion did not appear enthusiastic about performing when he or she was being positioned outside to enter the arena, that lion was omitted for that performance in favor of an enthusiastic lion, based on the trainer or presenter’s assessment. The same four lions participated in both performances the evening of the study. The same head trainer and assistant who presented the lions also did most of the daily feeding, training, and animal care. All of these lions clearly recognized and readily approached people they knew, but some lions preferred the company of specific people. For example, the adult male, Zeus, preferred interacting with women. The high level of training and the relaxed disposition of these lions when around people made them ideal subjects for this study.

The two Thermochrons that were recovered from Yoris provide extra confidence in the efficacy of these loggers and this methodology of assessing core body temperature. Both loggers responded immediately to the ingestion of the cold water, and then returned to core body temperature in a similar manner. Additionally, both loggers were expelled from Yoris at the same time and cooled down at the same rate to the 15 °C lower reading of the model H loggers. Although all of these lions had continuous access to water, Yoris was the only lion to drink enough water when the loggers were high enough in the gastrointestinal tract to register a change in temperature of the loggers. A useful line of future research could elucidate the relationship between gastrointestinal thermodynamics and locations of loggers within the digestive tract. We did not continuously observe the lions for drinking behavior, but assumed the other cats probably drank water later when they still had ingested loggers. Those instances likely reflect when the loggers were too far down the gastrointestinal tract to register a change from drinking water.

There was a limit to how long the trainers and researchers could keep searching through the bedding for the Thermochrons, and we hypothesized at the time that it was most likely that unrecovered loggers were simply overlooked because of their small size and the large amount of bedding. Removal of the soiled bedding from the ground was postponed as long as possible to allow for searching for the loggers and there was a limit to how long cleaning could be delayed before other activities needed to commence. Hence, there is a possibility that one or more of the unrecovered loggers fed during this study may have been expelled after we stopped searching for them. The transit time for these types of body temperature loggers could be further researched during hot weather when minimal bedding is utilized and the loggers would be easier to find. Additionally, having an observer to continuously watch each lion for signs of defecation, starting when the lions first become active in the morning, could be useful.

Trethowan et al. [6] provided useful reference data on the core body temperatures of free-ranging lions over different seasons. On days when their lions fed, their 24 h maximum body temperature was an average of 0.2 °C higher than on days when they did not feed. This likely reflected the increased muscular thermogenesis caused by hunting, killing, and consuming the prey. Although Trethowan et al. [6] surgically implanted loggers that recorded body temperatures at 5 min intervals; they did not report acute changes or diurnal rhythms, but concentrated on daily averages which fit the goals of that study. Although surgically implanted body temperature loggers can have certain advantages, they also need to be surgically removed. Feeding miniature loggers to lions can be useful for short-term applications with minimal impact on the welfare of the subject. However, the data loggers passing from the lions in less than 24 h can be a limitation. For example, Mortyola and Lanthier [1] based their analysis on deviation from the 24 h mean value.

There was no detectable increase in body temperature that could be attributed to the performances. Although not quantified, the lions spent much of each performance sitting on their pedestals waiting to be cued to perform the behaviors that resulted in being given a meat reward. Casual observations indicate that more energetic behaviors that contributed to muscle thermogenesis were performed when lions were playing with members of their group, interacting with lions in neighboring groups, soliciting attention from trainers walking by their exercise pens, or being transferred between pens and cages prior to and after a performance. The trend for body temperature to increase when Geta and Tantor’s loggers passed (09:00–09:45) occurred when the lions became active after the shutters on their dens were opened and they had access to their exercise pen. This temporary increase in body temperature was likely due to muscular thermogenesis associated with locomotor activity (e.g., [2]) resulting from exogenous activity in the vicinity of their exercise pens.

The temperature readings from inside the dens of Group 3 and Zeus need to be interpreted with caution. Because the lions had access to their dens, the Thermochrons had to be mounted on the wall or bars just outside the dens (about 0.5 m above the floor) so the lions could not reach the loggers. However, the loggers were still inside the flaps that were closed during the night. As a result, these readings indicate the air temperature was increased 3 to 5 degrees over ambient, but the sensors were not under the directional radiant heaters that warmed a portion of the cage. A benefit of this radiant zone heating is that the lions and tigers could regulate their microenvironment. They could move into a warmer area or a cooler area as they preferred. They could also burrow down into their bedding or snuggle with other cats within their group.

Due to the cold conditions and need for security, the sides of the lorries (their dens) were closed after the last performance and not opened again until daily activities started at 08:00. One direct observation of the lions (Group 3) was attempted at night by opening up the side of the den (lorry) wide enough to look in. The cats were all initially lying in the deep straw bedding, but as the side was lifted, the young lions all got up to see what was happening, and they aroused the other cats. Because opening the sides of the lorries just a small amount disturbed the cats, opening the lorries at night for behavioral observations was discontinued. The researchers could hear the lions quiet down in less than a minute. Regardless, the body temperature recordings indicate there was no sign of hypothermia in these lions.

Similarly to this study, Nevill et al. [5] fed Thermochron temperature loggers that recorded body temperature at 5 min intervals to circus tigers. Those tigers were being transported between venues and the goal was to look for signs of hyperthermia or hypothermia during hot and cold conditions. The authors reported a slight increase in body temperature during the loading process that was likely the result of the tigers being active, and independent of ambient temperature. All of those transports were relatively short and involved extraneous activities that complicated examination of those data for diurnal rhythms, except for one tiger. Transport of tiger 1, circus A, was completed at 23:23 and the tiger was not disturbed until 08:15. A slight decrease in body temperature throughout the night and into the morning is visible in their Figure 1, which is similar to the pattern reported in the lions in this study.

## 5. Conclusions

Feeding miniature temperature loggers imbedded in meat to lions was very simple. The challenge was in finding the loggers after they passed, and we recovered 70%. There was a significant diurnal effect, with core temperatures of 37.95 ± 0.42 °C at 15:50, and 36.81 ± 0.17 °C at 06:50 the next day, which were significantly different (t-test, t = 8.09, df = 6, *p* < 0.0003.). The mean duration for the time of passage was 22 ± 2.69 (h ± SD), with a range of 19.2 to 27 h. There was no evidence of hyperthermia or hypothermia because the lions had an abundance of bedding and could easily move under or away from the radiant heaters that were directed at an area in each den.

## Figures and Tables

**Table 1:** Information about the African lions (*Panthera leo*) who were fed body temperature loggers.

Name	Sex	Age	Loggers Found	Performed	Group	Pen Size (m^2^)	Den Area (m)
Wanda	F	8.0	One	Yes	1	200	12.3 × 2.2
Rose	F	7.7	One	Yes	1	200	12.3 × 2.2
Mery	F	7.7	None	No	1	200	12.3 × 2.2
Barbie	F	5.4	None	Yes	1	200	12.3 × 2.2
Denise	F	2.5	None	No	2	200	5 × 2.5
Geta	F	2.4	One	No	2	200	5 × 2.5
Zeus	M	8.2	Two *	Yes	Single	150	3 × 2.3
Iride	F	2.3	One	No	3	200	12 × 2
Tantor	M	2.3	One	No	3	200	12 × 2
Yoris	M	2.3	Two	No	3	200	12 × 2
Niko	M	2.3	None	No	3	200	12 × 2

* Two loggers were recovered from Zeus, but a young boy traveling with the circus held a magnet that he was playing with up against one of Zeus’s loggers to see if it was magnetic and accidentally erased the data.

## References

[1] Mortola J.P., Lanthier C. (2004). Scaling the amplitudes of the circadian pattern of resting oxygen consumption, body temperature and heart rate in mammals. Comp. Biochem. Phys. A.

[2] Refinetti R.G.J. (2018). Kenagy, G.J. Circadian rhythms of body temperature and locomotor activity in the antelope ground squirrel, *Ammospermophilus leucurus*. J. Therm. Biol..

[3] Reid K. (2019). Assessment of circadian rhythms. Neurol. Clin..

[4] Koch C.E.B., Leinweber B.C., Drengberg C., Blaum H.O. (2017). Interaction between circadian rhythms and stress. Neurobiol. Stress.

[5] Nevill C.H., Friend T.H., Toscano M.J. (2004). Survey of transport environments of circus tigers (*Panthera tigris*). J. Zoo Wildl. Med..

[6] Trethowan P., Fuller A., Haw A., Hart T., Markham A., Loveridge A., Hetem R., du Preez B., Macdonald D.W. (2016). Getting to the core: Internal body temperatures help reveal the ecological function and thermal implications of the lions’ mane. Ecol. Evol..

[7] Adam D., Johnston S.D., Beard L., Nicholson V., Lisle A., Gaughan J., Larkin R., Theilemann P., Mckinnon A., Ellis W. (2016). Surgical implantation of temperature-sensitive transmitters and data loggers to record body temperature in koalas (*Phascolarctos cinereus*). Aust. Vet. J..

[8] Bradshaw M.J., Keeling A.S., Bloomsmith M.A., Maple T.L. (2007). Environmental effects on the behavior of zoo-housed lions and tigers, with a case study of the effects of a visual barrier on pacing. J. Appl. Anim. Welf. Sci..

[9] Kiley-Worthington M. (1990). Animals in Circuses and Zoos: Chirons’ World.

[10] Krawczel P.D., Friend T.H., Windom A. (2006). Stereotypic behavior of circus tigers: Effects of performance. Appl. Anim. Behav. Sci..

[11] Nevill C.H., Friend T.H. (2006). A preliminary study on the effects of limited access to an exercise pen on stereotypic pacing in circus tigers. Appl. Anim. Behav. Sci..

[12] Nevill C.H., Friend T.H., Windom A.G. (2010). An evaluation of exercise pen use by circus tigers (*Panthera tigris tigris*). J. Appl. Anim. Welf. Sci..

